# Association of Metabolic Syndrome with the Cardioankle Vascular Index in Asymptomatic Korean Population

**DOI:** 10.1155/2015/328585

**Published:** 2015-07-27

**Authors:** Su-Hyun Nam, Sung-Goo Kang, Yun-Ah Lee, Sang-Wook Song, Jun-Seung Rho

**Affiliations:** ^1^Department of Family Medicine, St. Vincent's Hospital, College of Medicine, The Catholic University of Korea, Suwon 442-723, Republic of Korea; ^2^Health Promotion Center, St. Vincent's Hospital, College of Medicine, The Catholic University of Korea, Suwon 442-723, Republic of Korea

## Abstract

*Aim*. Metabolic syndrome is characterized by a cluster of atherosclerotic cardiovascular risk factors. The cardioankle vascular index (CAVI) reflects arterial stiffness and may be used as an indicator of atherosclerotic cardiovascular disease. In this study, we investigated the association of CAVI with metabolic syndrome. *Methods*. A total of 1,144 adults were included in this study. We measured CAVIs and examined blood samples to identify metabolic syndrome according to WHO Asia Pacific criteria and NCEP-ATPIII criteria. AST, ALT, *r*-GTP, BUN, creatinine, high sensitivity C-reactive protein, and uric acid were also measured. *Results*. CAVI values were significantly higher in subjects with metabolic syndrome than those without metabolic syndrome and increased according to the number of metabolic syndrome components present. Subjects with high fasting blood sugar levels or high blood pressure showed high CAVI values. Multiple regression analysis showed that age, sex, diastolic blood pressure, and uric acid were independent predictors of CAVI. *Conclusion*. Subjects with metabolic syndrome had high CAVIs, which indicated arterial stiffness, and were closely associated with an increase in the number of metabolic risk factors. The individual risk factors for metabolic syndrome have the synergistic effect of elevating arterial stiffness in asymptomatic Korean population.

## 1. Introduction

Metabolic syndrome is an abnormal metabolic state caused by a combination of arteriosclerotic risk factors such as abdominal obesity, dyslipidemia, glucose intolerance, and hypertension and is related to insulin resistance [1]. The national nutrition survey in Korea from 2007 to 2010 showed that the prevalence of metabolic syndrome in Korean adults aged over 30 years was 31.9% in men and 29% in women. Due to the fact that the lifestyle and food habits of the Korean people have become westernized and that the population has aged, metabolic syndrome continues to increase gradually in Korea [2]. Metabolic syndrome is associated with early vascular alterations, such as increased arterial stiffness and vascular endothelial cell injury, and increases the morbidity rate of type 2 diabetes mellitus and the mortality rate of cardiovascular disease [3, 4].

Pulse wave velocity (PWV) can be measured easily in noninvasive ways and has been considered a predictor of cardiovascular disease, reflecting vascular injury and arterial stiffness [5, 6]. Among these measures of PWV, brachial ankle PWV is seriously affected by hemodynamic variables such as blood pressure and autonomic nerve function, while the cardioankle vascular index (CAVI) is not influenced by blood pressure but reflects the stiffness of all blood vessels, including the aorta, the femoral artery, and the tibial artery [7]. CAVI, when measured with high accuracy, can be used as a more reliable indicator of arterial stiffness and the health of the arteries as they pertain to arteriosclerosis [7].

Nakamura reported that among 109 male and female adults who had undergone coronary angiography there was a positive relationship between the number of blood vessels with coronary artery stenosis and CAVI and a meaningful correlation between the severity of coronary atherosclerosis and the increase of CAVI [8]. It was suggested that CAVI can be used as a predictor of cardiovascular disorders in people with risk factors for cardiovascular disorders.

There is one study that showed the association between metabolic syndrome and CAVI in Chinese population. But there is a distinct lack of research on the usefulness of CAVI as an indicator of arterial stiffness in Korean metabolic syndrome patients. Research is needed to determine whether CAVI can be used as a good early predictor of cardiovascular disease and whether there is a relationship between CAVI and risk factors for cardiovascular disorders in metabolic syndrome. Thus, in this study, we investigate the relationship between CAVI and metabolic syndrome in Korean men and women.

## 2. Methods

### 2.1. Subjects

From January 2011 to January 2012, 1,144 male and female adults who had undergone a complete physical examination at a hospital in Gyeonggi-do were recruited. Eight who had a history of cancer, heart disease, and peripheral vascular disease were excluded.

### 2.2. Ethics Statement

This study was implemented in accordance with ethical and safety guidelines upon the approval of the Institutional Review Board in The Catholic University of Korea, St. Vincent's Hospital (IRB approval number UC 13RISI 0010). The study was exempted from the written informed consents of participants because we reviewed the health screening data and medical record retrogradely. The IRBs approved this consent procedure.

### 2.3. Physical Measurements and Blood Test

Weight and height were measured to one decimal place in kilograms and centimeters, respectively. Body mass index was calculated by dividing weight (kg) by the value of the square of height (cm^2^). Waist circumference (WC) was measured in centimeters at the midsection from the lower rib to the upper crista iliaca, while subjects were standing with their feet 30 cm apart, exhaling comfortably. Blood pressure was measured with an automatic blood pressure monitor while subjects were seated after having rested for 20 minutes. In the morning after subjects had fasted overnight, blood samples were drawn from the brachial veins. Fasting plasma glucose (FPG), triglycerides (TG), total cholesterol (TC), high-density lipoprotein cholesterol (HDL-C), aspartate transaminase (AST), alanine transaminase (ALT), gamma-glutamyl transpeptidase (r-GTP), blood urea nitrogen (BUN), creatinine (Cr), high sensitivity C-reactive protein (hs-CRP), and uric acid (UA) were also measured.

### 2.4. Measurement of CAVI

CAVI was measured noninvasively using a Vasera VS-1000 system. While subjects were resting in the supine position, cuffs were wrapped around both their upper arms and ankles. Electrocardiographic electrodes were attached to the upper arms, and a microphone was placed on the sternal angle to measure blood pressure and record an electrocardiogram and a phonocardiogram. After around 5 minutes, volume waveforms and the other data were printed from an embedded printer. PWV was calculated by dividing the directly measured length from the heart to the artery of ankle joint by the sum of the arrival time interval of the brachial pulse wave and the pulse wave of the ankle joint and the time from the closing sound of the aortic valve to the notch of the brachial pulse wave. From this value, the values of the brachial systolic blood pressure (SBP) and diastolic blood pressure (DBP) were applied to the formula of the stiffness parameter *β*. CAVI, based on the formula of stiffness parameter *β*, reflects the stiffness of the aorta, femoral artery, and tibial artery and is not influenced by blood pressure. CAVI was calculated using the following formula [5]:

(1) $$\begin{array}{c} \text{CAVI}=a\left[\left(\frac{2\rho }{\Delta P}\right)\times \ln\;\left(\frac{P_{s}}{P_{d}}\right){\text{PWV}}^{2}\right]+b \end{array}$$

(*P*_*s*_ is SBP; *P*_*d*_ is DBP; Δ*P* = *P*_*s*_ − *P*_*d*_; *ρ* is blood density; and *a* and *b* are constants).

### 2.5. Definition of Metabolic Syndrome

Metabolic syndrome was defined according to the criteria suggested in the report of the NCEP-ATPIII in 2005 and the Asia Pacific criteria. These criteria include the presence of any three of the following five risk factors [9, 10].

Criteria of metabolic syndrome are as follows:

1. abdominal obesity: WC of 90 cm or greater in men and 85 cm or greater in women;
2. TG: TG levels of 150 mg/dL (1.69 mmol/L) or greater or being on medicine to treat higher TG levels;
3. HDL-cholesterol: <40 mg/dL (1.04 mmol/L) in men, <50 mg/dL (1.29 mmol/L) in women, or being on medicine to treat lower HDL-cholesterol;
4. blood pressure: 130/85 mmHg or greater or being on medicine to treat hypertension;
5. glucose: 100 mg/dL (6.1 mmol/L) or greater or being on medicine to treat hyperglycemia.

### 2.6. Statistical Analysis

The data were analyzed using the Statistical Package for Social Sciences (SPSS) version 12.0 (SPSS Inc., Chicago, USA). The continuous variables in each of the two groups were compared using the* t*-test. We carried out analysis of covariance (ANCOVA) after adjusting for age in the association between CAVI and metabolic syndrome and performed multiple linear regression analysis to evaluate factors which affected CAVI. *P* < 0.05 was regarded as statistically significant.

## 3. Results

### 3.1. Characteristics of the Subjects

Of the 1,144 subjects, 372 (32.7%) had metabolic syndrome, 770 (63.7%) did not have metabolic syndrome, and 507 (69%) were men. Subjects in the metabolic syndrome group were older and had greater BMIs, WCs, SBP, DBP, FPG, TG, TC, AST, ALT, Cr, hs-CRP, and UA levels but had lower HDL-C levels than subjects in the nonmetabolic syndrome group. The characteristics of subjects are shown in Table 1; *P* < 0.001 was regarded as statistically significant.

### 3.2. Relationship between CAVI and Metabolic Syndrome Components

**Figure 1:**
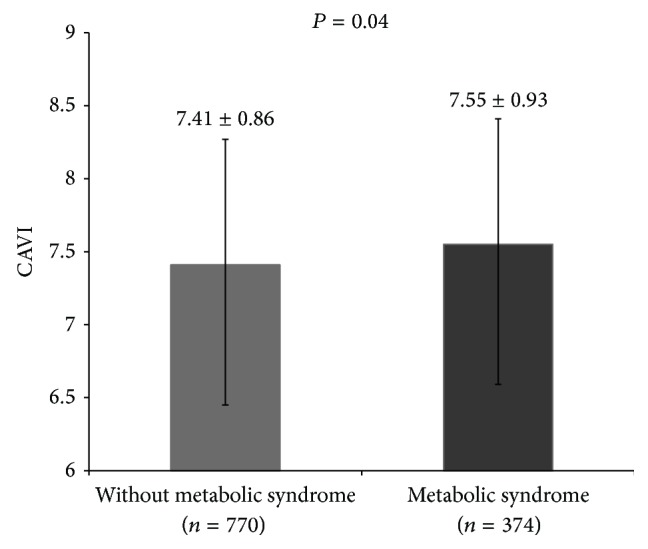
Comparison of cardioankle vascular index (CAVI) between subjects with and without metabolic syndrome. *P* values were calculated by ANCOVA and age-adjusted CAVI.

**Figure 2:**
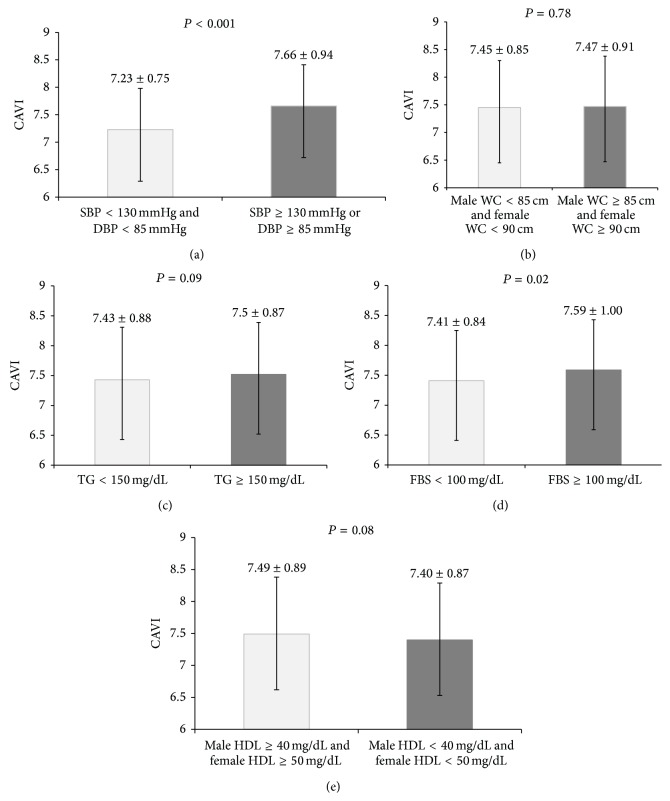
The relation between cardioankle vascular index (CAVI) and the component of metabolic syndrome. *P* values were calculated by ANCOVA and age-adjusted CAVI.

**Figure 3:**
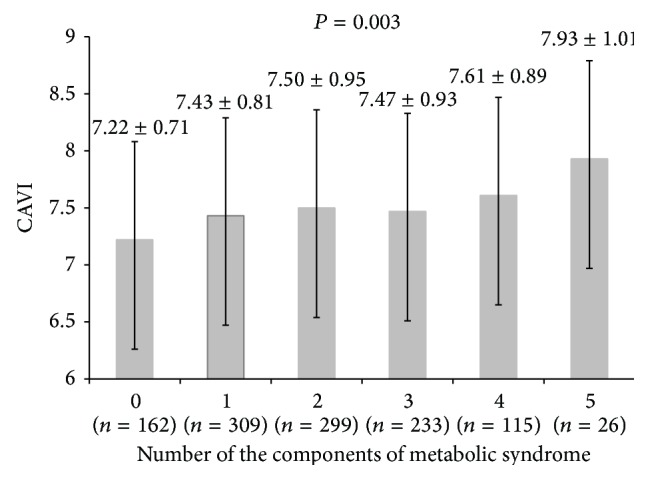
The relation between cardioankle vascular index (CAVI) and the number of components of metabolic syndrome. *P* values were calculated by ANOVA and age-adjusted CAVI.

The average value of CAVI was 7.55 ± 0.96 in the metabolic syndrome group, which was significantly higher (*P* = 0.04) than that of the nonmetabolic syndrome group (7.41 ± 0.86, Figure 1). CAVI values were statistically significantly higher in subjects with metabolic syndrome than those without metabolic syndrome after adjustment for sex, age, and the other factors (*β* = 0.143, *P* = 0.04). After analyzing the influence of the components of metabolic syndrome on CAVI, we found that subjects in the metabolic syndrome group whose SBP values were 130 mmHg or greater or whose DBP values were 85 mmHg or greater had higher CAVI values than subjects in the nonmetabolic syndrome group (*P* < 0.001, Figure 2). The group in which WCs exceeded 95 cm in men and 85 cm in women had slightly higher CAVI values than the control group, but the difference was not statistically significant (*P* = 0.78, Figure 2). In the group whose TG levels were 150 mg/dL or greater, higher CAVI values were observed than in the control group, but the difference was not statistically significant (*P* = 0.09, Figure 2). Higher CAVI values also were measured in the group whose FPG values were 100 mg/dL or greater, than in the group whose FPG values were 100 mg/dL or less, but the difference was not statistically significant (*P* = 0.02, Figure 2). CAVI values were slightly higher in the group with HDL-C levels less than 40 mg/dL in men and 50 mg/dL in women than in the control group, but the difference was not statistically significant (*P* = 0.08, Figure 2). After having analyzed the correlation between CAVI and the number of components of metabolic syndrome, we discovered that CAVI was significantly greater in the group which had more components (*P* = 0.003, Figure 3), and this relationship was linear (*F*(1,1138) = 18.180, *P* = 0.001).

### 3.3. The Factors That Affect CAVI

After performing multiple linear regression analysis, using risk factors for metabolic syndrome which can potentially influence CAVI, we found that age (*P* < 0.001), sex (*P* < 0.001), DBP (*P* = 0.04), and UA (*P* = 0.001) were independent predictors of CAVI (Table 2).

## 4. Discussion

The risk factors for coronary artery disease include old age, dyslipidemia, hyperglycemia, obesity, and hypertension, among others [4]. Except for age, these factors are consistent with the components of metabolic syndrome. As a result, since metabolic syndrome may increase the risk of coronary artery disease, detecting and managing cardiovascular disease risk factors early in people with metabolic syndrome are very important in preventing cardiovascular disease due to atherosclerosis.

PWV, as an index which reflects arterial stiffness and blood vessel injury, is a strong predictor of cardiovascular disease morbidity and mortality [5, 6]. CAVI is derived from stiffness parameter *β* theory and can be determined by measuring PWV and blood pressure. CAVI is not affected by blood pressure at the time of measurement and reflects arterial stiffness more precisely than brachial ankle PWV [7]. Thus, in this study we examined the relationship between the components of metabolic syndrome and CAVI as an index of arterial stiffness.

The study of Shirai et al. showed that, in adults aged 20 to 70 who received an annual health check and had no coronary risk factors, there was a direct association between CAVI and Age. CAVI increases with age almost linearly from 20 to 70 years in males and females. Also, CAVI of men is higher than that of women in almost all age groups [11]. In this study, after performing multiple linear regression analysis, using risk factors for metabolic syndrome which can potentially influence CAVI, we found that age and sex were independent predictors of CAVI.

In this study, CAVI values were found to be greater in the metabolic syndrome group than in the control group and were highly significant even when adjusted for age and sex. Also, in the group whose subjects had at least one component of metabolic syndrome, compared with the group whose subjects did not have metabolic syndrome, CAVI was statistically significantly higher and increased linearly with the number of metabolic syndrome components. These results are consistent with the results of previous studies which investigated the relationship between CAVI and metabolic syndrome [12, 13]. Metabolic syndrome components interrelate and cluster in various combinations, and they may promote increases in arterial stiffness and cardiovascular disease risk factors [12, 13]. CAVI values were influenced and elevated by the existence and increase of each metabolic syndrome component, even when the number of metabolic syndrome components was below the diagnostic criteria of metabolic syndrome [3]. Therefore, since metabolic syndrome components interact organically, metabolic syndrome components need to be managed to regulate arterial stiffness and prevent cardiovascular disease.

Among the components of metabolic syndrome, the elevation of SBP or DBP had a significant relationship with the elevation of CAVI. After having examined these relationships through multiple linear regression analysis, we found that DBP was an independent predictor of CAVI. The study of Takaki et al. showed that, in adults aged 30 to 80 who had undergone coronary angiographies, there was no direct association between CAVI and SBP or DBP [14]. The study of Ibata et al. showed that there was no significant relationship between CAVI and SBP in the case of normotensive patients [15]. However, in the study of healthy male and female adults of Kadota et al., simple regression analysis conducted with adjustment for age and gender showed that CAVI was related to SBP and DBP [16]. Furthermore, multiple linear regression analyses conducted with adjustment for several variables indicated that SBP was an independent predictor of CAVI. In the study of hemodialysis patients and type 2 diabetes patients of Shirai et al., CAVI had a minor association with SBP and no association with DBP [7]. In the study of hypertensive patients of Okura et al., CAVI had a minor association with SBP. CAVI was not influenced by blood pressure but was influenced by long-term hypertension status. Okura observed that CAVI could not be used to evaluate the long-term risks of arterial stiffness [17]. Arterial stiffness also is an independent predictor of the progression of hypertension in normotensive adults [18]. The elevation of CAVI resulting from the increased tension in arterial walls reflects the increase of arterial stiffness. Thus, the degree of elevation of CAVI may correspond to the degree of atherosclerotic change. A very high CAVI may indicate that the atherosclerotic process already is well underway [8].

Various hypotheses about the mechanism whereby metabolic syndrome influences arterial stiffness have been suggested. First, metabolic syndrome is associated with sympathetic nerve hyperactivity. The increase of tension in sympathetic nerves inhibits artery distensibility and increases the stiffness of blood vessels [19, 20]. The peptide adiponectin secreted by adipose tissue is associated with insulin sensitivity, is protective to blood vessels, and shows antiatherosclerotic and anti-inflammatory effects [21]. A decrease in adiponectin secretion results in the increase of arterial stiffness by promoting insulin resistance. In metabolic syndrome, the bioavailability of nitric oxide is decreased, endothelial cell dysfunction is promoted, and cytokines associated with various inflammatory processes are secreted and become involved in inflammatory reactions. The molecular components of these abnormal pathological states interact with each other. Hyperplasia of vascular smooth muscle cells, hypertrophy, and collagenesis are promoted, and arterial stiffness increases [22, 23].

In this study, increased FPG was among the metabolic syndrome components associated with a significant increase in CAVI. Ohnishi et al. reported that brachial ankle PWV increases with increasing FPG [24]. Significantly higher CAVI values were measured in type 2 diabetes patients. Some reports have shown that HbA1C is a significant risk factor for the increase of CAVI [15, 16]. These results are consistent with previous reports indicating that diabetes affects the PWVs of central elastic arteries selectively [25]. Diabetic patients are exposed to hyperglycemia in the long term, and advanced glycation end products (AGEs) are generated in their bodies by the nonenzymatic glycation of matrix proteins. AGEs contribute to cardiovascular complications such as increased atherosclerosis and vascular stiffness [26, 27].

One interesting finding in this study was that UA was significantly elevated in the metabolic syndrome group and was an independent predictor of the elevation of CAVI. The increase of serum UA has a significant association with metabolic syndrome and is a risk factor for cardiovascular disease [28, 29]. UA causes oxidative stress and endothelial cell dysfunction, is associated with blood vessel and systemic inflammatory reactions, and finally can lead to cardiovascular disease [30]. Sun et al. have reported that higher UA levels were detected in metabolic syndrome patients and that the increase of UA correlated with brachial ankle PWV [31]. Liu et al. reported that there was a significant relationship between CAVI and UA among persons with metabolic syndrome [13]. Nagayama et al. also reported that high serum uric acid is associated with increased CAVI in healthy Japanese subjects [32]. Sun et al. explained that the proinflammatory properties of UA could increase PWV by being partially related to the chronic inflammatory status seen at diagnosis of metabolic syndrome [31]. Therefore, this study has provided additional clues about the elevation of UA as a risk factor for cardiovascular disease.

This study has a few limitations. First, since this study was cross-sectional, we could not deduce the temporal order of the onset of metabolic syndrome and the elevation of CAVI. Second, as patients who came to the health promotion center of a general hospital were targeted, there could have been a selection bias. Third, we lacked some information associated with metabolic syndrome and arteriosclerosis, such as that regarding smoking, drinking, exercise, and family history.

Despite these limitations, this study contributes to the early assessment of cardiac disease by showing that metabolic syndrome and its components have a significant association with increased CAVI as a predictor of arterial stiffness in asymptomatic Korean population. In the future, a clinical trial will be needed to investigate whether CAVI decreases when metabolic syndrome components are improved, and a cohort study will be needed to investigate the relationship between CAVI and the onset of metabolic syndrome, to establish the risk of elevated CAVI in metabolic syndrome more clearly.

## Figures and Tables

**Table 1:** Clinical characteristics of subjects without and with metabolic syndrome.

	Nonmetabolic syndrome	Metabolic syndrome	*P* value
Age (year)	55.08 ± 8.28	56.43 ± 8.78	0.01
Sex			0.001
Male	507 (64.3%)	282 (35.7%)	
Female	263 (74.1%)	92 (25.9%)	
BMI (kg/m^2^)	23.18 ± 2.60	25.88 ± 2.62	<0.001
WC (cm)	82.93 ± 6.59	90.59 ± 6.08	<0.001
SBP (mmHg)	123.34 ± 14.54	131.91 ± 14.95	<0.001
DBP (mmHg)	74.25 ± 9.55	79.79 ± 10.41	<0.001
FBS (mg/dL)	92.06 ± 13.12	101.06 ± 16.52	<0.001
TG (mg/dL)	99.84 ± 52.64	193.17 ± 104.20	<0.001
TC (mg/dL)	202.97 ± 38.22	208.45 ± 35.38	0.02
HDL-C (mg/dL)	52.31 ± 28.48	40.53 ± 7.77	<0.001
AST (IU/L)	21.51 ± 8.91	23.19 ± 10.59	0.008
ALT (IU/L)	22.04 ± 14.02	29.48 ± 20.46	<0.001
Cr (mg/dL)	0.85 ± 0.20	0.87 ± 0.17	0.10
UA (mg/dL)	5.38 ± 1.38	5.91 ± 1.36	<0.001
CRP (mg/dL)	0.16 ± 0.48	0.23 ± 0.35	0.10

*P* values were calculated by *t*-test or chi-square test.

Values are presented as mean ± SD or number (%).

BMI, body mass index; WC, waist circumference; SBP, systolic blood pressure; DBP, diastolic blood pressure; FPG, fasting plasma glucose; TG, triglycerides; TC, total cholesterol; HDL-C, high-density lipoprotein cholesterol; AST, aspartate transaminase; ALT, alanine transaminase; Cr, creatinine; CRP, C-reactive protein; UA, uric acid.

**Table 2:** Multiple linear regression between CAVI and laboratory parameters.

Variables	*β*	*P* value
Age (year)	0.015	<0.001
Female	−0.233	<0.001
BMI (kg/m^2^)	−0.004	0.87
SBP (mmHg)	0.002	0.41
DBP (mmHg)	0.009	0.04
FBS (mg/dL)	−0.002	0.24
TG (mg/dL)	−0.001	0.09
HDL-C (mg/dL)	−0.001	0.54
UA (mg/dL)	0.064	0.001
WC (cm)	−0.017	0.06
AST (IU/L)	0.002	0.70
ALT (IU/L)	0.002	0.31
Number of the components of metabolic syndrome	0.117	<0.001

CAVI, cardioankle vascular index; BMI, body mass index; SBP, systolic blood pressure; DBP, diastolic blood pressure; FPG, fasting plasma glucose; TG, triglycerides; HDL-C, high-density lipoprotein cholesterol; UA, uric acid; WC, waist circumference; AST, aspartate transaminase; ALT, alanine transaminase.
